# Reading therapy strengthens top–down connectivity in patients with pure alexia

**DOI:** 10.1093/brain/awt186

**Published:** 2013-07-23

**Authors:** Zoe V. J. Woodhead, William Penny, Gareth R. Barnes, Hilary Crewes, Richard J. S. Wise, Cathy J. Price, Alexander P. Leff

**Affiliations:** 1 Wellcome Trust Centre for Neuroimaging, University College London, 12 Queen Square, London, WC1N 3BG, UK; 2 Department of Clinical Neurosciences, Royal Free Hospital and University College Medical School, NW3 2QG, London, UK; 3 Computational, Cognitive and Clinical Neuroscience Laboratory, Division of Experimental Medicine, Hammersmith Hospital Campus, Imperial College London, London, W12 0NN, UK; 4 Institute of Cognitive Neuroscience, University College London, 17 Queen Square, London, WC1N 3AR, UK; 5 Department of Brain Repair and Rehabilitation, Institute of Neurology, University College London, UK, WC1N 3BG, UK

**Keywords:** alexia, stroke, reading disorders, connectivity, magnetoencephalography

## Abstract

This study tested the efficacy of audio-visual reading training in nine patients with pure alexia, an acquired reading disorder caused by damage to the left ventral occipitotemporal cortex. As well as testing the therapy’s impact on reading speed, we investigated the functional reorganization underlying therapy-induced behavioural changes using magnetoencephalography. Reading ability was tested twice before training (t1 and t2) and twice after completion of the 6-week training period (t3 and t4). At t3 there was a significant improvement in word reading speed and reduction of the word length effect for trained words only. Magnetoencephalography at t3 demonstrated significant differences in reading network connectivity for trained and untrained words. The training effects were supported by increased bidirectional connectivity between the left occipital and ventral occipitotemporal perilesional cortex, and increased feedback connectivity from the left inferior frontal gyrus. Conversely, connection strengths between right hemisphere regions became weaker after training.

## Introduction

Pure alexia is an acquired reading disorder that spares central language abilities. Orthographic knowledge (the rules governing how words are built from letter combinations) is intact, as evidenced by sparing of both spelling and writing ability; however, degraded input to the orthographic system causes slow reading, characterized by an inability to process whole words efficiently (Binder and Mohr, 1992; Behrmann *et al.*, 1998; Patterson and Lambon-Ralph, 1999; Cohen *et al.*, 2004; Leff *et al.*, 2006). Patients usually only report their reading impairment, but there is good evidence that pure alexia is not really ‘pure’ as sensitive neuropsychological assessments have revealed co-occurring deficits for non-linguistic stimuli (Mycroft *et al.*, 2009; Starrfelt *et al.*, 2009). Unlike normal readers, patients with pure alexia take considerably more time to read long words than short words; an effect widely considered diagnostic and known as the word length effect (Warrington and Shallice, 1980; Leff *et al.*, 2001). This has a detrimental impact on patients’ quality of life, preventing them from being able to return to work, read letters or emails, or simply read for pleasure (Behrmann and McLeod, 1995).

Pure alexia is almost always caused by damage to the ventral occipitotemporal cortex (the area of cortex running from occipital cortex to the temporal lobe via the fusiform or lingual gyri) and associated white matter (Damasio and Damasio, 1983; Binder and Mohr, 1992; Leff *et al.*, 2006). Efficient word-form processing is clearly associated with this left-lateralized ventral stream, although debate continues over whether visual word recognition results from the function of neurons tightly tuned to familiar letter combinations within the mid-fusiform gyrus (Dehaene *et al.*, 2005; Dehaene and Cohen, 2011); or is dependent on interactions within a more distributed network that includes ventral occipitotemporal cortex as a key node (Patterson and Lambon-Ralph, 1999; Price and Devlin, 2003, 2011).

There have been many case studies of rehabilitation in pure alexia (Moyer, 1979; Tuomainen and Laine, 1991; Daniel *et al.*, 1992; Arguin and Bub, 1994; Lott *et al.*, 1994, 2010; Behrmann and McLeod, 1995; Seki *et al.*, 1995; Beeson, 1998; Maher *et al.*, 1998; Rothi *et al.*, 1998; Friedman and Lott, 2000; Sage *et al.*, 2005; Ablinger and Domahs, 2009; Lacey *et al.*, 2010); but to date, no group-level studies have been published and there is no generally accepted rehabilitation approach in current clinical use. Attempts to train compensatory reading strategies, such as kinaesthetic reading (tracing the letters to use the intact writing ability to facilitate word recognition), have had positive effects (Lott *et al.*, 1994, 2010; Seki *et al.*, 1995; Maher *et al.*, 1998; Sage *et al.*, 2005). However, the maximum reading speeds achievable using this technique are likely to be limited by the patient’s writing speed, and may actively prevent the recovery of the whole-word recognition strategy that affords rapid and efficient reading in skilled readers (Coslett *et al.*, 1993). For this reason, we chose to investigate whether patients can benefit from restitutive training of whole-word reading. Given the preservation of auditory perception of language in patients with pure alexia, we reasoned that cross-modal therapy (hearing and seeing words at the same time) might help retrain visual word form recognition through paired associate learning (Holcomb and Anderson, 1993) and mass practice.

Efforts to retrain the impaired whole-word reading ability have been reported in at least three previous case studies. Friedman and Lott (2000) employed a simple oral naming task, with brief presentation durations and feedback. Sage *et al.* (2005) used an errorless-learning approach, where the patient was first trained by presentation of written words on flash cards, which the experimenter initially read aloud; then the patient was required to repeat the word five times. Ablinger and Domahs (2009) used a two-stage approach: first the patient performed an audio-visual verification task on single words (similar to the task used in the present study), and then they performed an oral naming task with feedback. All three studies demonstrated that whole-word training improves word reading ability and decreases the tendency towards letter-by-letter reading. Two of the studies (Friedman and Lott, 2000; Sage *et al.*, 2005) found that the training effects were item specific, whereas Ablinger and Domahs (2000) observed generalization to novel words. Hence, there is evidence to suggest that the whole-word approach is effective, but the issue of generalization remains an open question.

The two competing models of word recognition could be used to suggest a number of different hypotheses regarding the mechanisms underlying whole-word reading rehabilitation. In terms of Dehaene’s localist model, training may strengthen or rebuild the local combination detectors, either in perilesional cortex or the mid-fusiform of the contralateral hemisphere. Alternatively, interactive models of reading place an emphasis on the connections between regions, in terms of which rehabilitation may result from stronger bottom-up connections from visual cortex, or stronger top-down connections from multimodal language areas.

The first aim of the present study was to test the efficacy of intensive, cross-modal word recognition training in a group of nine patients with pure alexia. Because the therapy was directed at improving whole-word reading (i.e. suppressing letter-by-letter reading) we predicted that training effects would be observed as a reduction in the word length effect. The second aim was to investigate the neural mechanisms of reading training, using Dynamic Causal Modelling (DCM) for magnetoencephalography (MEG) to identify changes in reading network connectivity after training. In a recent study we used DCM for MEG to investigate reading network connectivity (Woodhead *et al.*, 2012) and found that reading real words, compared to meaningless ‘false font’ stimuli, evokes feedback activity from the left inferior frontal gyrus to left ventral occipitotemporal cortex within the first 200 ms of word processing. This feedback connectivity was interpreted as playing a facilitatory role in word recognition in the ventral occipitotemporal cortex, similar to the feedback effects from frontal cortex observed during object recognition (Bar *et al.*, 2006; Kveraga *et al.*, 2007). Given this observation, we predicted that cross-modal training may serve to strengthen the feedback connections from the left inferior frontal gyrus to visual association cortex.

## Materials and methods

### Procedure

**Figure 1:**
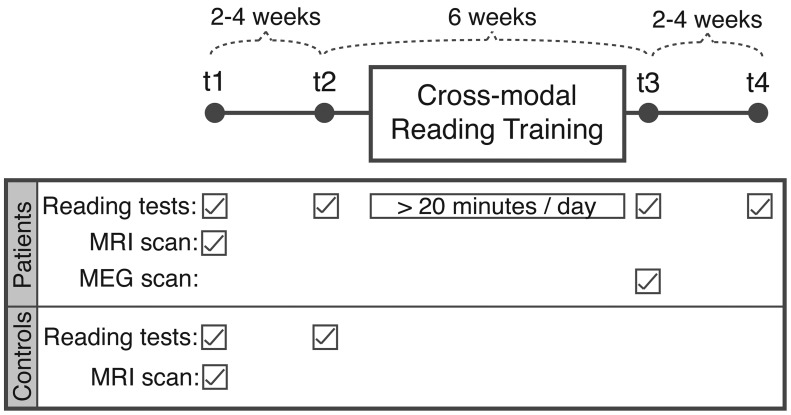
Schematic diagram of research protocol over four time points (t1 to t4).

A repeated-measures design was used, with four time points, t1 to t4 (Fig. 1). All participants completed reading assessments in two baseline sessions (t1 and t2) spaced by an interval of 2 to 4 weeks. After this, the patients took part in 6 weeks of reading training using custom-written software. The patients were reassessed immediately after completion of the training (t3), and at a follow-up session 2 to 4 weeks later (t4). The control subjects took part in t1 and t2 sessions only. In addition to the reading assessments, the patients had a structural MRI scan and neuropsychological assessment at t1, and an MEG scan, post-therapy only, at t3.

### Participants

Nine patients (three female, mean age 63 years, range 32–77 years) participated in the behavioural aspects of the study, but due to contraindications, not all patients were able to participate in the imaging. Eight patients (omitting Patient P6) participated in the MEG scanning. Structural MRI was performed in eight patients (omitting Patient P1).

Demographics for all nine patients are listed in Table 1. The inclusion criteria for patients were: (i) a self-reported reading impairment; (ii) left occipital or occipitotemporal focal brain injury; and (iii) a minimum of 6 months since brain injury. Reading tests before training began confirmed that all patients had three-letter word reaction times and word length effects at least two standard deviations larger than the control population average, consistent with the diagnosis of pure alexia. Exclusion criteria included: (i) impaired speech production, speech comprehension or writing (to rule out those with ‘central’ alexia); (ii) a premorbid history of neurological or psychiatric illness; or (iii) evidence of visual neglect or visuospatial processing deficits. Patients with visual field deficits were not excluded as these are commonly associated with the pure alexia syndrome.

**Table 1:** Demographic information for the patient group

Patient	Age at injury (y)	Time since onset (y, m)	Sex	Visual fields (sparing)	Cause of lesion	3-letter word RT (ms)	WLE (ms/letter)
P1	33	8, 11	M	RHH (1°)	Haemorrhage	953	202
P2	64	2, 6	M	Normal	Infarct	920	83
P3	67	9, 6	F	RHH (1°)	Infarct	1515	491
P4	45	8, 7	M	RHH	Head injury	1244	308
P5	47	6, 5	M	RSQ	Infarct	791	57
P6	75	0, 6	F	RIQ	Infarct	1289	269
P7	67	7, 2	M	RIQ	Infarct	723	26
P8	69	8, 11	F	RIQ	Infarct	782	72
P9	61	8, 1	M	RSQ	Infarct	1168	49

RT = reaction time; WLE = word length effect; RHH = right homonymous hemianopia; RIQ = right inferior quadrantanopia; RSQ = right superior quadrantanopia.

Sparing = the extent of spared visual field expressed in degrees from the vertical meridian; if not stated, there was no sparing.

Nine age- and gender-matched controls (three female, mean age 62 years, range 30–80 years) were recruited to provide normative data for the pretraining reading assessments. A paired-subjects *t*-test confirmed that there was no statistically significant difference in age between the two groups [*t*(8) = 1.3, *P* = 0.23]. The control participants were in good general health, with normal or corrected-to-normal eyesight and no history of neurological or psychiatric disorders. All participants were right handed, spoke English as a first language and had a normal history of reading development.

All participants gave informed written consent in accordance with the Declaration of Helsinki. Participant information was provided in both audio and written forms where necessary. Research procedures were approved by the National Hospital of Neurology and Neurosurgery and Institute of Neurology Joint Medical Ethics Committee.

### Reading training

The reading training was computer-based and self-administered. Patients were given a laptop computer loaded with the training software and were instructed to use it for at least 20 min a day over the 6-week training period. Actual usage was recorded by the software.

The software cycled through training and testing blocks. In training blocks, single words were presented centrally on the screen for 500 ms while the spoken word was played simultaneously. Spoken word recordings were acquired in an anechoic chamber by a single female speaker, and the sound files were normalized for mean amplitude. Patients were required only to attend to the word pairs. The brief presentation duration was employed to suppress a letter-by-letter reading strategy: although 500 ms is a long presentation time for control subjects, it is a short time for patients with pure alexia (Saffran and Coslett, 1998) and substantially shorter than the oral word naming speed of any patient tested here (Table 3). The inter-onset interval was 2 s. After 15 word pairs, the software proceeded to a testing block. In each testing trial, patients were presented with a written word taken from the preceding training block that was paired with a spoken word. In half of the testing trials the written and spoken words matched, and in the other half they were different. The patient was required to make a same/different response by button press. The advantage of the same/different discrimination task over oral naming was that it allowed automated recording of the patients’ performance without the need for voice recognition software.

Task difficulty was altered according to performance by varying the similarity between the written and spoken words in the ‘different’ test trials. Each word of the training list was paired with three spoken words, ranging in difficulty from easy to hard. The two words of the ‘easy’ pair (e.g. ‘food/hate’) did not share the same initial letter, and shared few or none of the remaining letters (0.24 letters were shared per word on average across all word pairs). The ‘medium’ word pair (‘food/fill’) did share the same initial letter, and shared 1.22 letters per word on average. The ‘hard’ word pair (‘food/foot’) shared the same initial letter and at least one other letter, with an average of 3.50 shared letters per word.

The ‘easy’ word pair was used for the initial presentation of a word in a ‘different’ testing trial; if this was answered correctly, the next ‘different’ trial for that word would use the ‘medium’ word pair and so on. Only one correct or incorrect response on a ‘different’ trial was sufficient to progress up to the harder level or return back down to the easier level respectively. In order to encourage patients to spend time on the therapy, a monetary reward of one pence per correct answer was awarded and displayed on a score counter in the bottom-left corner of the screen.

The training stimuli were selected from the Medical Research Council’s Psycholinguistic Database (Coltheart, 1981) and were between three and six letters in length. Short words were chosen so that the whole word could be perceived within one ocular fixation. They were split into two word lists (List A and List B), each containing 500 words; and were matched for letter length, syllable length, written frequency and imageability (confirmed by Mann-Whitney non-parametric tests, *P* > 0.4 in each case). Each patient was trained with one of the two word lists, and list allocation was counterbalanced across the group. The untrained list was subsequently used to investigate possible generalization effects of the lexical reading therapy. The two lists were approximately matched for part of speech (nouns: List A = 207/500 words, List B = 189; verbs: List A = 187, List B = 183; adjectives: List A = 63, List B = 83; other: List A = 43, List B = 45).

Orthographic similarity was not controlled when designing the word lists, but the degree to which the trained and untrained words shared the same letter combinations is a pertinent factor in interpreting the training results. To take an extreme example, if the two word lists shared no letter combinations in common, it would be impossible to distinguish whether faster reading speed for trained words than untrained words had been driven by learning at the level of whole-word representations or sub-lexical representations. On the other hand, if the two-word lists had a high degree of orthographic overlap, a specific improvement in trained words would be most likely to result from training at the whole-word level. To this end, orthographic similarity between the two word lists was assessed at the level of bigram and trigram frequency. Each word was decomposed into its constituent bigrams and trigrams (e.g. house: bigrams = [ho], [ou], [us] and [se]; trigrams = [hou], [ous] and [use]). Frequencies were counted with which each bigram or trigram appeared again in the same word list (within-list bigram frequency and within-list trigram frequency) and in the other word list (between-list bigram frequency and between-list trigram frequency). These values were then divided by the number of bigrams or trigrams in the word (e.g. house: four bigrams and three trigrams) to give within- and between-list bigram and trigram frequencies for each word, independent of word length. A 2 × 2 repeated measures ANOVA, with the levels orthographic segment length (bigram versus trigram) and search list (within-list versus between-list), and the between group factor training list (List A versus List B), showed no significant difference in orthographic frequency within or between word lists [i.e. no main effect of search list, *F*(1,998) = 2.7, *P* = 0.101]. In other words, there was no evidence that the two word lists were orthographically distinct.

### Reading assessment

Word, letter and text reading speed and accuracy were assessed in all participants at all time points.

Three versions of the word-reading test with non-overlapping stimuli were used to ensure that the same words were not repeated in sequential time points. Each version of the test consisted of 128 trials, with equal numbers of 3, 4, 5 and 6-letter words to allow calculation of the word length effect. Half of the words came from word List A and half from List B in order to test item specificity of training effects. One-way ANOVAs confirmed that there were no significant differences in Kucera-Francis written frequency (Kucera and Francis, 1967) or imageability between the word sets in different test versions, letter lengths or word lists (*P* > 0.3 in all cases).

The word reading test was presented using E-Prime software (Schneider *et al.*, 2002). Words were presented centrally on a screen in black, lower case, size 36 Arial font on a grey background. Participants were instructed to read the word aloud as fast as possible. A voice-key was used to detect the latency of the response onset. The words remained on screen until a response was detected. Practice trials were administered before testing to allow the participant to become accustomed to speaking clearly into the microphone. Trials affected by malfunction of the voice-key (for example, by erroneously detecting non-speech sounds, or by omitting to detect valid responses) were excluded from the analysis. This affected 3.2% of all trials. To calculate average reaction times, the mean reading speeds for 3, 4, 5 and 6-letter words were calculated, and responses more than two standard deviations away from the mean were excluded to reduce positive skew. Word length effects were calculated by taking the average increase in reading speed per additional letter. To calculate accuracy, trials read correctly scored 1; errors or omitted words scored 0; and verbal false-starts or self-corrected errors scored 0.5.

The letter-reading test used two testing sheets of letter stimuli from the Birmingham Object Recognition Battery (Riddoch and Humphreys, 1993). Letters were written in capitalized, size 42, Times New Roman font, in black ink on white paper. Each sheet contained 18 letters evenly spaced across the page. Participants were asked to read the letters out loud as fast and accurately as possible. Each sheet was administered separately, and the time taken to read all the letters was recorded manually with a stopwatch. The accuracy scoring system was the same as in the word reading test.

Text reading was tested with two short pieces of narrative text at each time point. Whereas the patients were asked to read the texts as fast as possible, control participants were instructed to read at a natural reading pace, in order to provide ‘normal’ reading speeds for comparison with the patients. Eight different text narratives were created, and tested in a small group of volunteers to ensure that average reading speeds did not differ significantly between texts. The texts ranged from 87 to 94 words in length and were based on newspaper articles, modified to remove any highly infrequent words or names. Average reading speed in words per minute was calculated for each text. Accuracy was recorded using the same error-rating scheme as the letter and word reading tests.

### Structural magnetic resonance imaging and lesion mapping

A T_1_-weighted structural brain image with whole-brain coverage was acquired for each patient using a Philips Intera 3.0 T MRI scanner and an eight-array head coil. Lesions were outlined by hand using tools from the FMRIB Software Library (Smith *et al.*, 2004; Woolrich *et al.*, 2009). The resulting binary lesion images were smoothed with a 2 mm full-width at half-maximum Gaussian kernel and inverted to produce a cost-function mask for registration to Montreal Neurological Institute (MNI) space using FMRIB’s Linear Image Registration Tool (Jenkinson and Smith, 2001; Jenkinson *et al.*, 2002). Cost-function masking ensures that lesioned areas are down-weighted to zero in the registration process (Brett *et al.*, 2001). The native-to-standard space transformation matrix was then applied to the smoothed lesion masks, and the resulting images from each subject were combined to produce a lesion overlay map. Detailed information of the patients’ lesion locations is available in Supplementary Table 1.

### Magnetoencephalography scanning procedures

The MEG data were acquired with a VSMMed Tech Omega 275 MEG scanner with an array of 275 axial gradiometers in software third gradient-mode. The sampling rate was 480 Hz with a 120 Hz anti-alias filter. Fiducial markers on the nasion and the left and right pre-auricular points were used to determine head location in the scanner.

There were two MEG runs, each containing 110 trials. Fifty trials contained words from the trained word list, 50 contained words from the untrained word list and 10 were familiar names (e.g. john, tim, sarah, etc) used as ‘catch trials’ in an incidental task to maintain the participants’ attention throughout the scan. Participants were instructed to read the words silently and press a button whenever they read a familiar name. Trained and untrained words were matched for word length, and did not differ significantly for word frequency or imageability.

Cogent software (www.vislab.ucl.ac.uk/cogent.php) was used to present trials in a pseudorandom order in the centre of a screen ∼50 cm in front of the participant. Words were presented in lower case, size 50 Helvetica font, in black on a grey background to minimize visual glare. They were between 3–6 letters in length and subtended less than 3.5 visual degrees either side of fixation. Words were presented for 1000 ms and separated by 1500 ms of central fixation.

### Magnetoencephalography analysis

#### Preprocessing and source localization

MEG data analysis was performed using Statistical Parametric Mapping 8 software (SPM8; Litvak *et al.*, 2011; http://fil.ion.ucl.ac.uk/spm) in MatLab 7.11 (The MathWorks Inc.). MEG data from catch trials were excluded from the analysis. Data from all sensors were high-pass filtered at 1 Hz. Eye-movement artefacts were removed using the Multiple Source Eye Correction method (Berg and Scherg, 1994), which involves fitting an equivalent current dipole at the location of each eyeball and subtracting the resulting source waveforms from the sensor data. Trained and untrained word trials were epoched from −100 to 1000 ms peri-stimulus time. The prestimulus time window was used for baseline correction. A low-pass filter at 30 Hz was applied, and the trained and untrained trials were averaged using robust averaging (Litvak *et al.*, 2011). Robust averaging is a form of robust general linear modelling (Wager *et al.*, 2005) used to down-weight outliers in the MEG data. Finally, the low-pass filter (30 Hz) was repeated to eliminate any high frequency noise introduced by the averaging process.

In order to visualize the M170 response for trained words, averaged MEG evoked responses to trained and untrained words were squared and summed over all sensors to calculate global field power for each condition, and for each subject. These plots were then averaged across the patient group to produce average global field power plots for trained and untrained words (separately). Although the control subjects did not participate in the training experiment, comparable MEG data were available from a group of eight age-matched controls from a previous study (Woodhead *et al.*, 2012). In this study, healthy controls read single words or visually-matched but meaningless false font stimuli. Stimuli were presented to the controls for 500 ms (rather than 1000 ms in the patients) with a presentation rate of one stimulus every 3 s. Otherwise, the MEG protocol and analysis were identical to the present study (Woodhead *et al.*, 2012).

Source localization was performed using variational Bayesian equivalent current dipoles (Kiebel *et al.*, 2008); a point-source localization method that uses a non-linear optimization algorithm to test the fit of a number of dipoles, with different prior distributions on their locations and moments, to sensor data at a particular point in time. The first step in this process was to identify the exact time point at which to fit the source dipoles to the sensor data. The M170 was chosen as it is a strong and reliable peak that was present in all participants, and is known to be related to orthographic processing (Rossion *et al.*, 2003; Maurer *et al.*, 2005). The input data for the variational Bayesian equivalent current dipoles analysis for each subject was the combined average of all trained and untrained words, so that the source localization was not biased towards one condition over the other. For each subject, the average response to all trained and untrained trials was calculated, and the M170 peak was identified in a semi-supervised manner. The resulting peaks had an average latency of 184 ms, with a range from 167 to 210 ms.

The next step was to select the set of dipole locations to use as spatial priors for the variational Bayesian equivalent current dipoles analysis. Previous work on word reading in healthy controls using the same methodology (Woodhead *et al.*, 2012) demonstrated that the M170 response to reading words (or visually-matched false font stimuli) was optimally fitted by a 6-source model with spatial priors in left and right occipital cortices (MNI coordinates±15 −95 2), ventral occipitotemporal cortices (±44 −58 −15) and inferior frontal gyri (±48 28 0). These sources were used in the present study.

**Figure 2:**
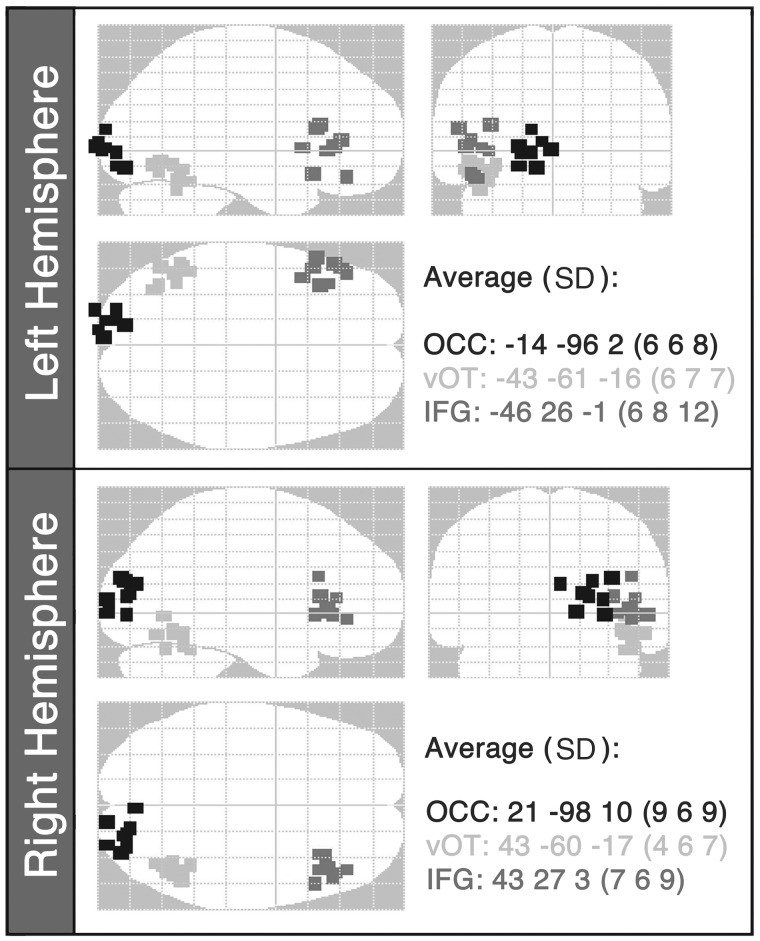
Optimal source locations from the variational Bayesian equivalent current dipole analysis for each subject plotted on a glass brain in MNI space. The starting points for the source locations were: occipital cortex: ±15 −95 2; ventral occipitotemporal cortex: ±44 −58 −15 14; inferior frontal gyrus: ±48 28 0. The average locations (with standard deviations, SD) of the winning source locations for the patient group are also reported. OCC = occipital cortex; vOT = ventral occipitotemporal cortex; IFG = inferior frontal gyrus (*n* = 8).

A single-shell forward model was used (Nolte, 2003). The variational Bayesian equivalent current dipoles algorithm varied the locations and moments of each dipole over 100 iterations to find the solution with the largest model evidence. For each iteration, a different starting location was selected at random for each source from a Gaussian distribution with standard deviation of 6 mm in each direction from the previous mean. The source locations with the highest model evidence for each subject (Fig. 2) were checked on the subject’s normalized T_1_-weighted structural brain image to ensure that: (i) they were consistent with their anatomical labels; (ii) no sources fell within the patients’ brain lesions (Supplementary Fig. 1); and (iii) no pair of sources was separated by a Euclidian distance <2 cm.

All patients except Patient P2 had damage to primary left visual cortex or its inputs, resulting in a right visual field defect (hemianopia or quadrantanopia). Despite this, it was possible to locate equivalent current dipole sources in perilesional cortex of the left occipital lobe. The response in the left primary visual cortex may be the result of interhemispheric transfer of visual information from the right occipital lobe, consistent with abundant homotopic interhemispheric connections in the visual system (Jarbo *et al.*, 2012). Alternatively, the influence of peri-lesional left occipital responses might be neglected in hemianopia or quadrantanopia (i.e. subconscious) but effective in driving higher level functions. These hypotheses require further investigation.

#### Dynamic causal modelling

The effect of training on effective connectivity within the reading network was assessed using DCM for MEG in SPM8 (David *et al.*, 2006; Kiebel *et al.*, 2006, 2009). DCM uses a biologically-informed, fully-generative approach, whereby the observed activity in each MEG source is modelled as a combination of signals arising from three layers of neurons: pyramidal cells, spiny stellate cells and inhibitory interneurons. The response rates and intrinsic connectivity patterns of the three layers are defined according to the neural mass model of Jansen and Rit (1995) and laminar organization described by Felleman and Van Essen (1991). The sensitivity of each source to its inputs, the connections between MEG sources, and the effects of stimulus-driven modulation of connection strengths can also be modelled. Model estimation identifies the values of the model parameters that maximize the fit of predicted neural activity to the observed data. The outputs of this process are estimated values for each model parameter, and a quantification of the overall model evidence (i.e. goodness of fit, taking model complexity into account). In practice, a number of models are estimated, each varying in the particular parameter of interest. When the model space (the number of estimated models) is relatively small, the estimated model with the strongest model evidence is taken as the winner (Bayesian model selection; Penny *et al.*, 2004). Alternatively, if the potential model space is large, it may be more appropriate to average the value of individual parameters across all estimated models (Bayesian model averaging; Penny *et al.*, 2010).

In the present study, DCM was used to find which connections within the network of six MEG sources were modulated by stimulus type (trained versus untrained words). Modulatory connections represent the gain on the connection strength according to modulation by trial type. A gain of 1 would indicate that the connection strength was equal for trained and untrained words, whereas a gain significantly greater/lower than 1 would indicate stronger/weaker connectivity for trained words relative to untrained words, respectively. The observed data to be modelled were the preprocessed MEG data from 1–200 ms after stimulus presentation. The subject-specific MEG source locations identified by the variational Bayesian equivalent current dipole analysis were used as spatial priors on the DCM source locations. An exogenous input was modelled in the left and right occipital cortex nodes at 60 ms peri-stimulus time.

In a six-source network, there are 30 different connections. If each connection can be modulated independently, there is a total of 2^30^ unique models, which is clearly too large to be computationally viable. Following Woodhead *et al.* (2012), the following connectivity rules were used to constrain the model space:

- Connections can be forward, backward or lateral, but not diagonal, e.g. from left occipital cortex to right ventral occipitotemporal cortex.
- Every forward or backward connection must be matched with an identical connection in the opposite hemisphere, e.g. if a connection exists from left occipital cortex to left ventral occipitotemporal cortex, there must also be a connection from right occipital cortex to right ventral occipitotemporal cortex.
- Every lateral connection must be matched with a reciprocal connection in the opposite direction, e.g. if a connection exists from left occipital cortex to right occipital cortex, there must also be a connection from right occipital cortex to left occipital cortex.

This reduced the number of independent connections to nine, making the total model space 2^9^, or 512. All 512 models were estimated per participant, each with a different combination of connections mediating trial-specific modulations.

In the final step, group-level Bayesian model averaging with random effects (Penny *et al.*, 2010) was used to average the modulatory effect on each connection across all estimated models and all participants. A non-parametric proportion test was used on each connection to evaluate whether the modulatory gain was significantly different to 1. This was done by taking 10 000 random samples from a Gaussian distribution based on the mean and standard deviation of the gain on each connection. If 90% of the samples were greater or smaller than 1, the connection was judged to be significantly stronger for trained or untrained words, respectively, at P > 0.9 (Richardson *et al.*, 2011; Seghier *et al.*, 2012; Woodhead *et al.*, 2012). This probability, P, is the Bayesian posterior probability of the connection being present given the neuroimaging data. This quantity can also be expressed as a Bayes factor, with a posterior probability of 0.9 corresponding to a Bayes factor of P/(1−P) = 9. This means that it is nine times more likely than not that the connection is present. We emphasize that P is not a classical *P*-value. In general there is no direct mapping from Bayes factors to *P*-values, but in the case of two-sample *t*-tests, for example, Bayes factors of nine correspond to ∼*P* = 0.002 (see Fig. 3 in Wetzels *et al.*, 2011).

**Figure 3:**
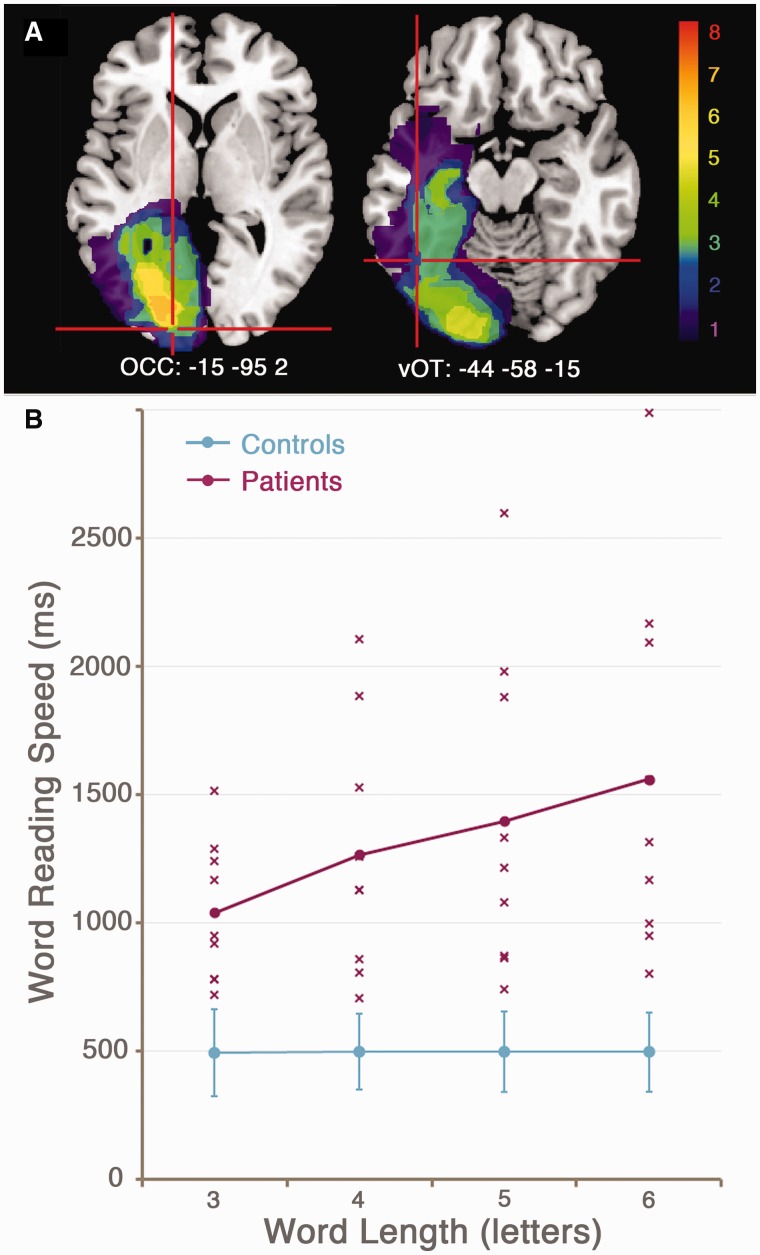
(**A**) Lesion overlay map for the patient group, demonstrating the area of maximal overlap in occipital white matter of the left hemisphere. Crosshairs indicate the location of the left occipital (OCC) and ventral occipitotemporal (vOT) spatial priors for the source localization analysis (*n* = 8). (**B**) Reading speed slopes demonstrating the effect of word length (3–6 letters) on reading latency for the patient group (pink) and control group (blue). Each cross represents data from an individual patient.

## Results

### Structural lesion mapping

MRI structural imaging data were available for eight patients. The patient group showed maximal lesion overlap in the white matter of the left occipital lobe (Fig. 3A). Seven patients (Patients P2, P3, P4, P5, P6, P8 and P9) had damage to the occipitotemporal portion of the fusiform gyrus and/or the white matter tract immediately above this area, including the inferior longitudinal fasciculus (Yeatman *et al.*, 2012). The remaining patient (Patient P7) had damage to the occipitotemporal portion of the lingual gyrus. Critically, none of the patients had lost all of the left ventral occipitotemporal cortex that is normally activated during reading. Therefore it was possible to identify perilesional ventral occipitotemporal cortex reading activations in all patients.

### Reading ability before training

Letter, word and text reading were tested at two baseline assessments before training (t1 and t2). Paired *t*-tests were applied to investigate potential practice effects. Letter reading speed showed a trend towards faster reading at t2 than t1 in the patient group only [*t*(8) = 1.9, *P* = 0.098]. This effect was attributed to a better understanding of the task demands at the second time point. Test–retest effects between t1 and t2 did not approach significance for word and text reading tests. Data were averaged over the two time points to create a more robust baseline measure.

Table 2 reports between-group comparisons (patients versus controls) of reading performance. The patients were significantly worse than controls at all measures except for letter reading accuracy, where both groups were at ceiling. The patients had significantly larger word length effects than the control subjects. Figure 3B demonstrates the word length effect as an increase in reaction times as word length increased. The word length effect closely correlated with reading speed for three-letter words (*R* = 0.86, *P* < 0.005) and single letters (*R* = 0.74, *P* < 0.05).

**Table 2:** Pretraining reading measures averaged over the two pretraining time-points (t1 and t2) for patient and control groups

Measure	Patients mean (SD)	Controls mean (SD)	*t*
Texts, speed (wpm)	63.4 (32.0)	174.2 (25.7)	8.1***
Texts, accuracy (%)	95.2 (3.7)	99.2 (0.7)	3.2**
Words, speed (ms/word)	1308 (512)	497 (78)	4.7***
Words, accuracy (%)	91.0 (9.3)	99.1 (0.6)	2.7*
Word length effect (ms/letter)	173 (157)	1.12 (9.4)	3.3**
Letters, speed (ms/letter)	775 (321)	370 (77)	3.7**
Letters, accuracy (%)	99.0 (2.0)	99.8 (0.3)	1.4

Standard deviations are given in brackets.

T-statistics are reported from paired-subjects *t*-tests comparing patients and controls.

Wpm = words per minute.

*P < 0.05; **P < 0.01, ***P < 0.001.

*n* = 9 in each group.

Figure 3B also shows the large variability in reading speed within the patient group. Patients were selected for participation according to the presence of a significant word reading deficit, defined as three-letter word reading speed and word length effect of at least two standard deviations (SD) from the control group mean (three-letter word reading speed: mean = 495.3 ms, SD = 84.3 ms, inclusion criterion = 663.9 ms; word length effect: mean = 1.1 ms/letter, SD = 9.4 ms/letter, inclusion criterion = 20.0 ms/letter). Even the least impaired patient (Patient P7) was well within these criteria, with a three-letter word reading speed of 723 ms, and a word length effect of 26 ms/letter.

### Training effects on reading ability

To evaluate training effects on word reading speed, data were averaged across t1 and t2 (pretraining) and compared with data at t3. A 2 × 2 × 4 repeated-measures ANOVA was computed, with factors Time (pretraining versus t3), Word list (trained versus untrained words) and Word length (3, 4, 5 or 6-letters). This showed significant main effects of word list [*F*(1,8) = 12.0, *P* < 0.01] and word length [*F*(3,24) = 8.6, *P* < 0.001]. There was also a significant time by list interaction [*F*(1,8) = 14.2, *P* < 0.001] and a significant time by list by word length interaction [*F*(3,24) = 4.1, *P* < 0.05]. *Post hoc* paired *t*-tests showed that trained words were read significantly faster than untrained words after training at t3 [*t*(8) = 4.5, *P* < 0.005]. On an individual level, all patients showed faster reading speeds for trained words than untrained words at t3 (Supplementary Fig. 2).

**Figure 4:**
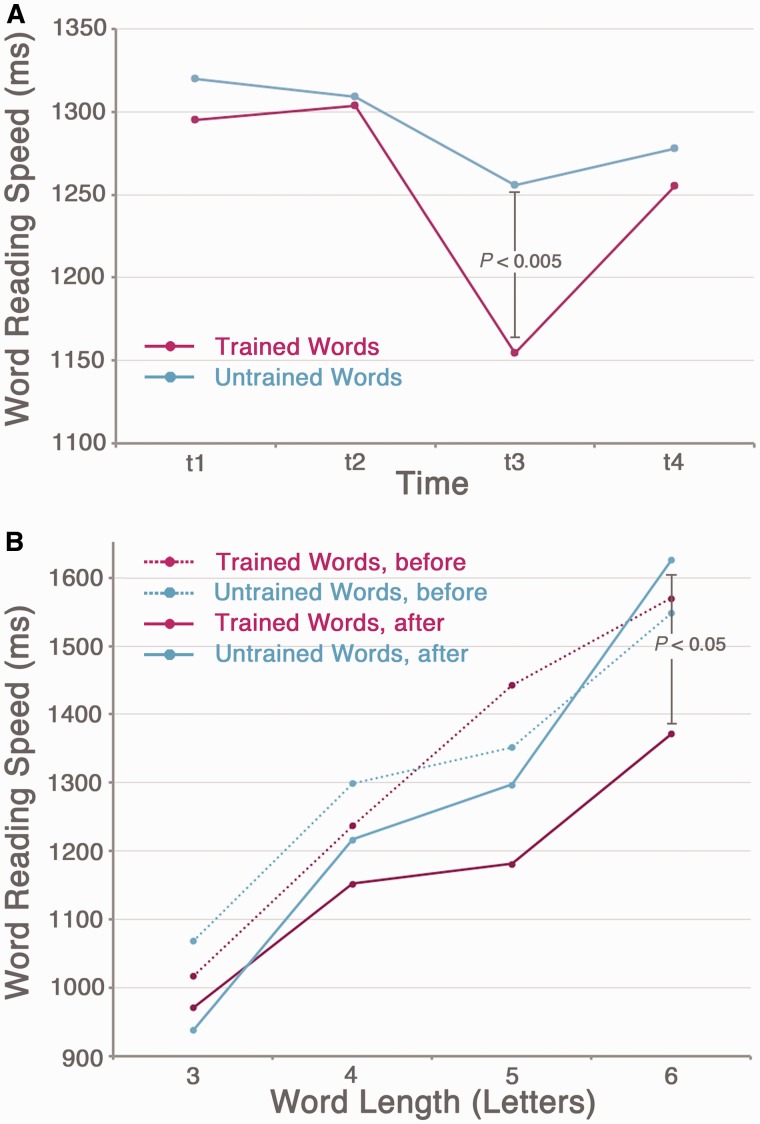
The effect of reading training on word reading speed. (**A**) Average word reading speed across all patients at each time point (t1–t4) for trained (pink line) and untrained (blue line) words. Training occurred between t2 and t3. (**B**) Reading speed slopes showing the effect of word length before training (the average of t1 and t2, dotted lines) and after training (t3, solid lines) for trained (pink) and untrained (blue) words. (*n* = 9).

Figure 4A shows the change in the patient group’s average word reading speed for trained and untrained words over time, collapsed across word length. The group average reading speed for trained words between t2 and t3 improved by 149 ms, a training effect of 11.5%. Figure 4B demonstrates word length effect slopes for trained and untrained words, before and after training. The greatest training effects were evident for longer words.

A repeated-measures ANOVA on word reading accuracy showed a significant main effect of time [*F*(1,8) = 6.9, *P* < 0.05; average trained word accuracy before training = 91.0%; at t3 = 93.3%], but the critical interaction of time with word list was not significant; both trained and untrained word reading accuracy improved after training. There were no significant effects of training on the letter or text reading measures. These results indicate that the training effect was specific to single word reading speed and did not generalize to other measures of reading ability.

### Influence of sub-lexical orthographical frequency

As described previously, the trained and untrained word lists were not significantly different in terms of their sub-lexical orthographical composition. However, it remains possible that reading speed of untrained words at t3 was influenced by sub-lexical orthographic similarity to trained words. In other words, if the training had worked by strengthening sub-lexical representations, it would be predicted that untrained items containing letter combinations that occurred frequently in the trained list would be read more quickly. The effect of sub-lexical orthographic frequency on untrained word reading speed was tested by calculating, for each untrained word, the number of times its constituent bigrams and trigrams appeared in the trained word list. The resulting bigram and trigram frequency counts were then divided by the total number of bigrams and trigrams within the word (respectively), resulting in a bigram frequency index and trigram frequency index for each untrained word (independent of word length). Partial correlations were computed separately for each of the nine patients, correlating untrained word reading speed at t3 and bigram frequency or trigram frequency, factoring out the effect of word length on reading speed (a total of 18 correlations). Correlations were weak, ranging from *R* = −0.26 to 0.24. On average, the correlation between reading speed and bigram frequency was *R* = 0.034, and between reading speed and trigram frequency *R* = 0.0056. No individual correlation reached significance. These results do not support the hypothesis that reading speed of untrained words was influenced by orthographic similarity to trained words.

### Reading ability at follow-up session

Word reading speed was assessed at a follow-up session between 2 to 4 weeks after completion of the training (t4), in order to test whether the effects of training were maintained over time. No further training or maintenance activities were performed between t3 and t4. A 2 × 2 × 4 repeated-measures ANOVA was performed, with factors Time (pretraining versus t4), Word list (trained versus untrained) and Word length. A main effect of word length was observed [*F*(1,8) = 10.3, *P* < 0.001], but there were no other significant effects or interactions. This indicates that the significant training effect on word reading speed observed at t3 was not maintained until t4.

### Training effects on network connectivity

MEG data were available in eight of nine patients (omitting Patient P6). To ensure that the behavioural effect of training on word reading speed was still significant in this reduced group, the 2 × 2 × 4 repeated-measures ANOVA with factors Time (pretraining versus t3), Word list (trained versus untrained) and Word length (3, 4, 5 or 6-letters) was repeated. As before, this showed significant main effects of word list [*F*(1,7) = 8.5, *P* < 0.05] and word length [*F*(1,7) = 6.6, *P* < 0.005], and a significant time by list interaction [*F*(1,7) = 10.5, *P* < 0.05]. The time × list × length interaction also approached significance [*F*(3,21) = 3.0, *P* = 0.054]. At t3, trained words were read faster than untrained words [*t*(7) = 4.1, *P* < 0.005].

**Figure 5:**
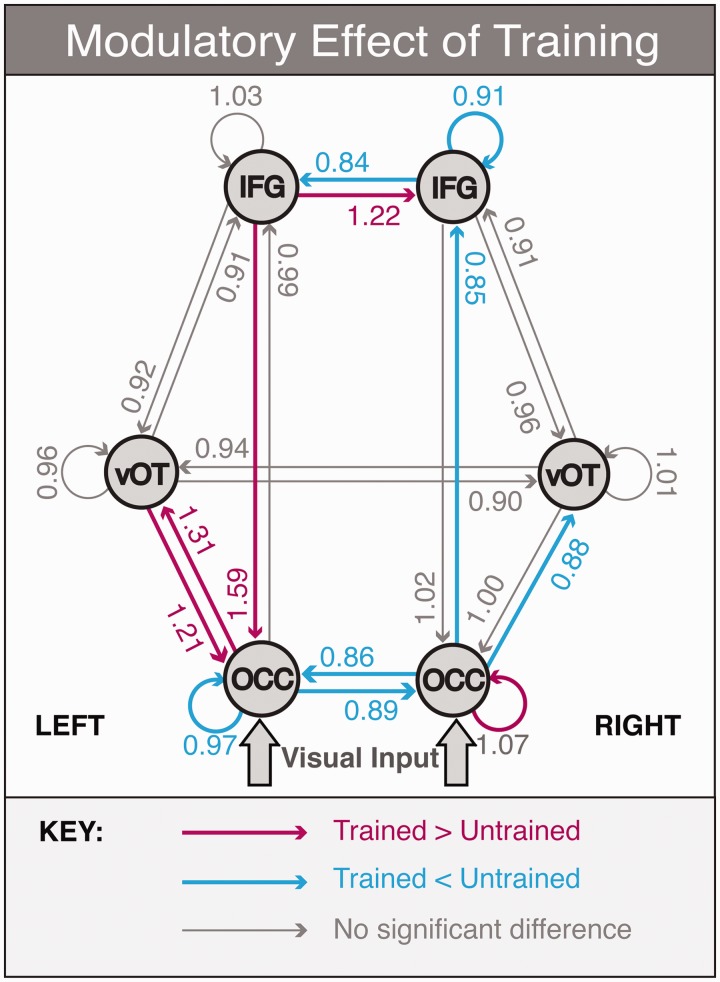
Results of the DCM analysis demonstrating the modulatory effects of stimulus type (trained versus untrained words at t3) on connection strengths. Numerical values represent the posterior mean of the gain on connection strength: values significantly greater or smaller than 1 indicate connections that are stronger for trained words (in pink) or stronger for untrained words (in blue), respectively. Significance threshold: P > 0.9 (*n* = 8). OCC = occipital cortex; vOT = ventral occipitotemporal cortex; IFG = inferior frontal gyrus.

The effects of training on the neural network for reading was assessed in the patient group at t3, immediately after completion of the reading training, using DCM for MEG. The results are shown in Fig. 5. Table 3 shows the posterior means and exceedance probabilities for connections that were above the Bayesian significance criterion of P > 0.9.

**Table 3:** Posterior means and exceedance probabilities for connections which were (A) significantly stronger for trained than untrained words (mean > 1) and (B) significantly weaker for trained than untrained words (mean < 1)

Connection	Posterior mean	Exceedance probability
(A) Trained > Untrained words		
Left OCC to left vOT	1.31	0.991
Left vOT to left OCC	1.21	0.988
Left IFG to left OCC	1.59	0.999
Left IFG to right IFG	1.22	0.970
Right OCC self-connection	1.07	0.991
		
(B) Trained < Untrained words		
Left OCC self-connection	0.97	0.914
Left OCC to right OCC	0.89	0.912
Right OCC to left OCC	0.86	0.962
Right OCC to right vOT	0.88	0.946
Right OCC to right IFG	0.85	0.989
Right IFG self-connection	0.91	0.926
Right IFG to left IFG	0.84	0.926

OCC = occipital cortex; vOT = ventral occipitotemporal cortex; IFG = inferior frontal gyrus.

### Stronger connections for trained than untrained words

There were five connections that were significantly stronger for trained than untrained words at the group level. These included the feedback connection from left inferior frontal gyrus to left occipital cortex; bidirectional connections between left occipital cortex and left ventral occipitotemporal cortex; the lateral connection from left inferior frontal gyrus to right inferior frontal gyrus; and the self-connection on the right occipital cortex source. In the DCM framework self-connections model the sensitivity of a region to its inputs (Kiebel *et al.*, 2007); therefore, a stronger self-connection implies that the right occipital cortex was more sensitive to trained than untrained words.

### Weaker connections for trained than untrained words

A number of connections were significantly weaker for trained than untrained words, and were largely symmetrical to connections that showed the opposite effect (trained > untrained). These included the bidirectional lateral connections between left and right occipital cortex nodes; the forward connections from right occipital cortex to right ventral occipitotemporal cortex and from right occipital cortex to right inferior frontal gyrus; the lateral connection from right inferior frontal gyrus to left inferior frontal gyrus; and the self-connections on the left occipital cortex and the right inferior frontal gyrus.

### Magnetoencephalography-evoked response fields

**Figure 6:**
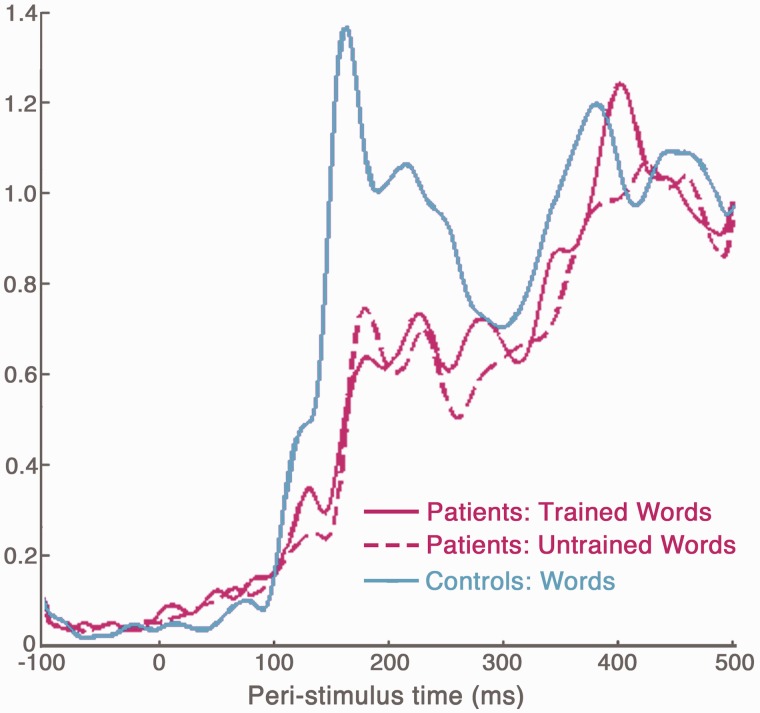
Global field power during word reading in the patient group (trained words, pink solid line; untrained words, pink dotted line) and a group of age-matched healthy controls (blue) using data from Woodhead *et al.* (2012).

Sensor space activity was visualized by calculating the global field power for each condition in each patient, and averaging across each group (Fig. 6). A within-subjects *t*-test compared the patients’ global field power amplitude for trained and untrained words at each time point. This revealed significantly stronger activation for trained words between 118–133 ms [peak difference at 127 ms, *t*(7) = 4.78, *P* < 0.005]. No other significant differences were observed.

Comparable MEG data for word reading were available for a group of age-matched healthy controls (Fig. 6, blue). Although we cannot compare the patient and control DCM results directly (because of the difference in stimuli and training), we were able to compare patient (trained words) and control global field power amplitude at each time point using a two-tailed independent samples *t*-test. Global field power amplitude differed significantly (*P* < 0.05, uncorrected for multiple comparisons) between the two groups at two time-windows: between 22–53 ms [patients > controls, peak difference at 42 ms, *t*(14) = 3.12, *P* < 0.01] and between 151–160 ms [controls > patients, peak difference at 157 ms, *t*(14) = −2.22, *P* < 0.05]. There were no significant differences between patients and controls after 200 ms (minimum *P-*value between 200–500 ms = 0.31).

## Discussion

### Behavioural effects of training

This is the first group-level study to report a positive effect of reading rehabilitation in patients with pure alexia. Critically, the lexical reading training was most effective for longer words, and hence reduced the word length effect, suggesting that the therapy worked by restoration of whole-word reading, rather than by improving the efficiency of a compensatory letter-by-letter reading strategy. If the latter had been the case, we would have observed a consistent improvement in reading speed across all word lengths; that is, a reduction in the intercepts but not the slopes of patients’ word length effect plots.

The training improvements did not generalize to novel, untrained words. Similar item-specificity has been observed in training case-studies by Lacey *et al.* (2010) and Friedman and Lott (2000). Lacey *et al.* (2010) tested the generalizability of the Multiple Oral Re-reading technique (Moyer, 1979), an approach that involves repetitive reading of a single piece of text. The authors showed that this technique improved text reading when the trained and probe texts shared a significant number of words, but that there was no generalization to probe texts that had no words in common with the trained text. The lack of generalization to text reading in the present study may reflect an insufficient overlap between the training corpus and the text reading stimuli. Friedman and Lott (2000) tested the efficacy of training that involved an oral naming task with immediate feedback, using brief presentation times to promote whole-word recognition. Their patient (Patient PW) showed improved reading accuracy for trained words but not untrained words. Furthermore, Patient PW was unable to learn to read pseudowords using this method. These findings imply that whole-word training requires access to existing lexical knowledge, either because it strengthens access to existing orthographic representations, or because formation of new orthographic representations is supported by feedback from intact phonological or semantic lexical representations. In either case, it is evident that future studies using a whole-word approach must carefully select an ecologically-valid training corpus in order to generalize to everyday reading situations.

The results also demonstrated that generalization of training effects was unaffected by sub-lexical orthographic frequency—in other words, untrained items containing letter combinations that had a high frequency in the training corpus were not read faster than untrained words with low frequency letter combinations. In the context of an existing model of word reading, the Local Combination Detector model (Dehaene *et al.*, 2005; Dehaene and Cohen, 2011) argues against the possibility that reading training works by strengthening or rebuilding letter combination detectors in the posterior fusiform gyrus that are damaged in pure alexia, and is more supportive of a word-specific effect driven by abstract representations higher up in the reading hierarchy.

The training effects were not well maintained over time. Reading speed was substantially improved immediately after training (t3), but began to revert towards baseline levels by the follow-up session 2 to 4 weeks later (t4). This is in keeping with evidence from the human expertise literature suggesting that deliberate practice is required to maintain peak performance (Ericsson *et al.*, 2006).

### Connectivity effects of training

Our anatomical reading network consists of three hierarchically organized levels, inferior frontal gyrus at the top, ventral occipitotemporal cortex in the middle and occipital cortex at the bottom, where sensory inputs enter via thalamic relay. According to Mumford (1992), the higher regions process more abstract (e.g. multimodal) features whereas those in the lower regions are more concrete (e.g. unimodal). In the predictive coding framework that builds on Mumford’s model (Friston, 2005), perception is dependent on feedback connections relaying abstract information down the hierarchy to meet bottom-up signals induced from sensory epithelium. Feed-forward connections code mismatches between sensory impressions and previous expectations, and are extinguished efficiently when predictions are at their most veridical. In light of this, the results from the DCM analysis supported our hypothesis that training would lead to stronger feedback connectivity in the left hemisphere (from inferior frontal gyrus to occipital cortex and from ventral occipitotemporal cortex to occipital cortex). The implication is that the cross-modal training increased the influence of higher-order processing in both the left inferior frontal gyrus and surviving left ventral occipitotemporal cortex over the lower-order visual cortex.

Previous studies have also suggested that feedback from the frontal cortex plays a role in facilitating visual processing, both in object recognition (Bar *et al.*, 2006; Kveraga *et al.*, 2007) and in word reading (Cornelissen *et al.*, 2009; Wheat *et al.*, 2010). Most compellingly, Lee and D’Esposito (2012) have recently demonstrated that transcranial magnetic stimulation of the left inferior frontal gyrus impairs performance on a visual working memory task; and by using a multivariate pattern analysis of concurrent functional MRI data, they showed that transient disruption of left inferior frontal gyrus is associated with decreased tuning of representations in extrastriate visual cortex. This compliments our data and suggests that the left inferior frontal gyrus has an important role to play in occipital cortical function irrespective of stimulus category.

It is interesting to note that a similar DCM analysis in healthy controls, comparing reading of written words to a false font baseline, observed a significant connection in the same time-window from the left inferior frontal gyrus to the left ventral occipitotemporal cortex, not the occipital cortex (Woodhead *et al.*, 2012). The stronger feed-forward connection for trained words between left occipital cortex and left ventral occipitotemporal cortex was unexpected. It may be that the orthographic processing here was only partially strengthened by therapy, with the presumed beneficial effect of the inferior frontal gyrus to occipital cortex connection magnifying the mismatch between these two levels. This interpretation is supported by the fact that although the therapy improved reading behaviour, it did not return to the level of the normal control subjects. The changes in connectivity described here probably represented either a partial restoration of the normal neural mechanisms of word recognition, or the adoption of a compensatory reading strategy. The reduction in word length effect after training seems to preclude the possibility of a serial letter-by-letter compensatory strategy, but this does not rule out the existence of alternative ‘whole-word’ recognition mechanisms that may offer faster and more efficient reading speeds.

One might expect to observe concomitant reduced feed-forward connectivity for trained versus untrained items between the same regions, but actually this was seen in the right hemisphere (occipital cortex to inferior frontal gyrus and ventral occipitotemporal cortex, Fig. 5). The connections between the inferior frontal gyrus are lateral connections according to anatomical criteria; however, the left inferior frontal gyrus is clearly more specialized for language, that is, it conveys more complex or abstract word knowledge derived from previous experience than its homologue. According to this functional definition it can be considered to be above the right inferior frontal gyrus in hierarchical terms, with trained items causing an increase in the strength of the left inferior frontal gyrus to right inferior frontal gyrus feedback (prediction) connection and a relative decrease in the feed-forward (prediction error) connection from right inferior frontal gyrus to left inferior frontal gyrus. There was no evidence to suggest that rehabilitation involved a compensatory increase in activity in the right ventral visual stream as previously proposed (Cohen *et al.*, 2004; Henry *et al.*, 2005); rather, the data support the idea that restoration of function in perilesional tissue around the damaged left ventral visual stream supported more efficient reading of trained items in our patients. Patients with extensive damage to ventral occipitotemporal cortex are likely to be more reading impaired than the participants in this study (Binder and Mohr, 1992). They may have to rely on the dorsal stream to support their residual reading ability (Seghier *et al.*, 2012), and thus may require therapy designed to optimize use of this route.

Lastly we will discuss therapy-associated changes seen within the lowest level of the reading network (occipital cortex). In DCM, self-connections dictate the sensitivity of a region to its inputs, whether they are forwards, backwards or lateral. We found increased sensitivity to trained words in the right occipital cortex region and the opposite (reduced sensitivity) in the left. We would have predicted both to increase, but one possible explanation is that the therapy is causing patients to focus their attention more on the initial letters of words, which project to the right hemisphere. For patients with a hemianopia there is more discriminatory information in the initial letters of a given word than in the last letters. Most patients with pure alexia have a hemifield disturbance of some sort (Leff *et al.*, 2001); in this study all did. These changes in self-connectivity were associated with a decoupling of the two occipital cortex regions for trained words. Decoupling of occipital cortices is associated with visual stimulation compared with rest (Nir *et al.*, 2006), but it is not clear why this effect should preferentially affect trained items.

Our aim was to examine how early orthographic processing was affected by pure alexia and influenced by training. In terms of time bins the 1–200 ms time-window was most appropriate because orthographic processing is associated with the M170, which is the main peak of neural activity associated with reading. As shown in Fig. 6, the amplitude of the M170 peak during reading was weaker in the patient group relative to age-matched, healthy controls, although this difference did not survive correction for multiple comparisons. There was no significant difference between patients and controls beyond 160 ms. In addition, a within-subjects comparison of global field power amplitude for trained words versus untrained words (in the patient group only) showed no significant differences beyond 200 ms. Indeed, when we extended the DCM time-window to 1–300 ms, we did not observe any additional effects of training. The question of why the later evoked components are normal in the patient group, despite the reduced amplitude of the M170 response is intriguing, but beyond the scope of the current paper.

In summary we used an audiovisual, cross-modal therapy to rehabilitate whole-word reading in a group of patients with pure alexia. Therapy effects were consistent across the group and were item-specific. Although the behavioural training effects were relatively small (11.5% improvement in word reading speed) and transient, we believe that, in rehabilitation terms, this is a promising first step that can be built upon by improving the training methodology. In addition, our study is novel in its aim to identify neural changes associated with this therapy-driven behavioural improvement. This approach is important in order to understand the mechanisms of functional reorganization that might be targeted in future rehabilitation studies. For instance, the increased feedback from the left inferior frontal gyrus observed for trained > untrained words raises the testable hypothesis that stimulation of this region (by transcranial direct current stimulation or similar methods) may enhance training effects in what is commonly accepted to be a highly treatment-resistant disorder (Leff and Behrmann, 2008). This change in feedback connectivity is in accord with the hypothesis that the training worked by making abstract, long-term word representations more veridical. In terms of models of word reading, our results demonstrate that top-down influences occur early in word processing (within the first 200 ms) and are modified by training. This is consistent with models of reading which explicitly propose an interaction of bottom-up and top-down processing, such as the interactive model of Behrmann and colleagues (1998) or Price and Devlin (2003, 2011).

## Supplementary Material

Supplementary Data

## Abbreviations

DCM: Dynamic Causal Modelling
MEG: magnetoencephalography
