# Supplementary material for: Reading therapy strengthens top–down connectivity in patients with pure alexia
Source: Brain. 2013 Jul 23;136(8):2579–91. doi: 10.1093/brain/awt186 (PMC3722354; doi:10.1093/brain/awt186)
Supplement: Supplementary Data [file supp_136_8_2579__index.html]

Reading therapy strengthens top–down connectivity in patients with pure alexia — Supplementary Data 

# Reading therapy strengthens top–down connectivity in patients with pure alexia

## 

files

**Files in this Data Supplement:**

- Supplementary Data - docx file
- Supplementary Data - jpg file
- Supplementary Data - jpg file
