# Supplementary material for: Reading therapy strengthens top–down connectivity in patients with pure alexia
Source: Brain. 2013 Jul 23;136(8):2579–91. doi: 10.1093/brain/awt186 (PMC3722354; doi:10.1093/brain/awt186)
Supplement: Supplementary Data [file supp_awt186_brain-2012-02024-File009.docx]

**Supplementary Data**

**Supplementary Figure S1**

Figure showing the location of left OCC and vOT dipoles for each patient, plotted on normalised structural brain images. Patients P1 and P6 are not represented here: P1 had contraindications to MRI scanning so no lesion image was available; P6 did not take part in the MEG scanning.

**Supplementary Figure S2**

Patients are ranked on the basis of reading speed at baseline, from the slowest (bottom of figure) to the fastest (top of figure). Bars indicate the change in reading speed (t2 – t3) in milliseconds, for trained words (pink) and untrained words (blue). All patients showed bigger gains (or smaller loses) for trained words than untrained words. There was a non-significant trend for bigger gains in the slower readers than the faster readers.

**Supplementary Table S1**

Further anatomical description of the patients’ lesions. Y=damaged; n=not damaged.

| Patient | Lesion volume  (cm^3^) | Striate cortex | Lingual gyrus | Fusiform gyrus | Interior temporal gyrus | Inferior longitudinal fasiculus | Corpus callosum | Other regions |
| --- | --- | --- | --- | --- | --- | --- | --- | --- |
| P2 | 17.3 | n | n | y | y | n | n | Second occipital gyrus |
| P3 | 74.7 | y | y | n | n | y | y |  |
| P4 | 143.8 | n | n | y | y | y | n | Ventral anterior temporal pole |
| P5 | 66.7 | y | y | y | y | y | y |  |
| P6 | 80.6 | y | y | y | y | y | n |  |
| P7 | 27.2 | y | y | n | n | n | n |  |
| P8 | 29.0 | y | y | y | n | y | n |  |
| P9 | 77.2 | y | y | y | n | y | n | Parahippocampal gyrus |
|  |  |  |  |  |  |  |  |  |
