# Supplementary figures and images for: Reading therapy strengthens top–down connectivity in patients with pure alexia
Source: Brain. 2013 Jul 23;136(8):2579–91. doi: 10.1093/brain/awt186 (PMC3722354; doi:10.1093/brain/awt186)

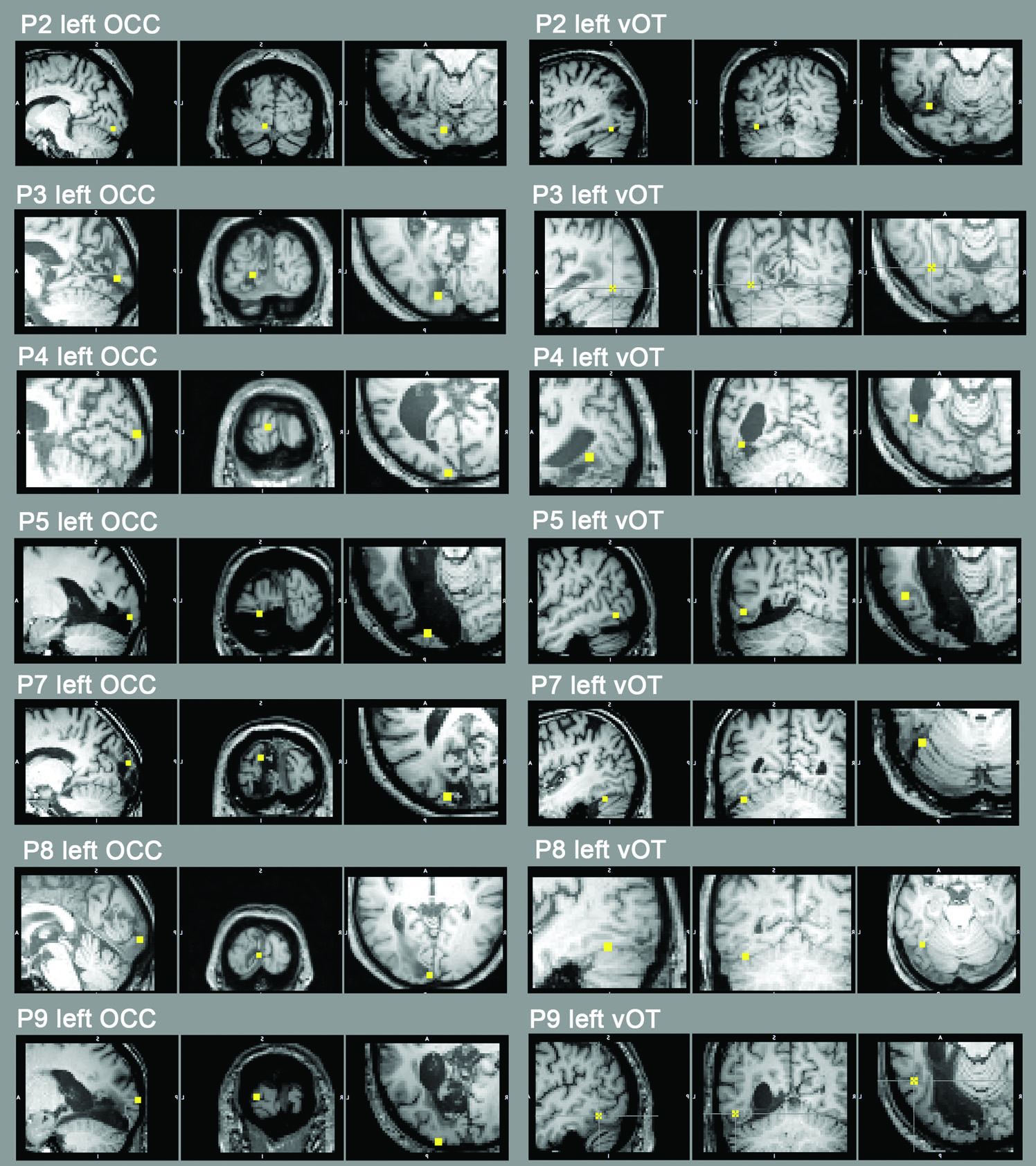

Supplement: Supplementary Data [file supp_awt186_brain-2012-02024-File010.jpg]

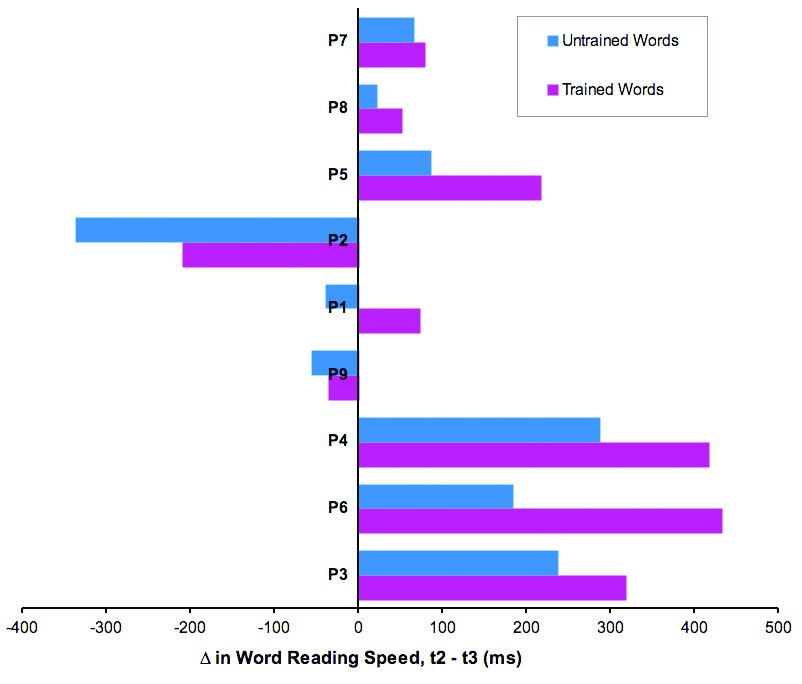

Supplement: Supplementary Data [file supp_awt186_brain-2012-02024-File011.jpg]
